# Impacts of land use at multiple buffer scales on seasonal water quality in a reticular river network area

**DOI:** 10.1371/journal.pone.0244606

**Published:** 2021-01-06

**Authors:** Zhimin Zhang, Fei Zhang, Jinglong Du, Dechao Chen, Weiwei Zhang

**Affiliations:** 1 College of Geography Science and Geomatics Engineering, Suzhou University of Science and Technology, Suzhou, Jiangsu, China; 2 College of Resources and Environment Science, Xinjiang University, Urumqi, Xinjiang, China; Northeastern University (Shenyang China), CHINA

## Abstract

The assessment and prediction of regional water quality are fundamental inputs to environmental planning and watershed ecological management. This paper explored spatiotemporal changes in the correlation of water quality parameters (WQPs) and land-use types (LUTs) in a reticular river network area. Water samples of 44 sampling sites were collected every quarter from 2016 to 2018 and evaluated for dissolved oxygen (DO), total phosphorus (TP), ammonia nitrogen (NH_3_-N), and permanganate index (COD_Mn_). A redundancy analysis (RDA) and stepwise multiple linear regression (SMLR) were applied to analyze the land-use type impacts on seasonal WQPs at five buffer scales (100, 200, 500, 800, and 1000 m). The Kruskal–Wallis test results revealed significant seasonal differences in NH_3_-N, TP, COD_Mn_, and DO. The area percentages of farmland, water area and built-up land in the study area were 38.96%, 22.75% and16.20%, respectively, for a combined total area percentage of nearly 80%. Our study showed that orchard land had an especially favorable influence on WQPs. Land-use type impacts on WQPs were more significant during the dry season than the wet season. The total variation explained by LUTs regarding WQPs at the 1 km buffer scale was slightly stronger than at smaller buffer scales. Built-up land had a negative effect on WQPs, but orchard and forest-grassland had a positive effect on WQPs. The effects of water area and farmland on WQPs were complex on different buffer scales. These findings are helpful for improving regional water resource management and environmental planning.

## Introduction

Water quality plays an increasingly important role in the industrial, agricultural and public health sectors [1]. However, water quality degradation is a worldwide phenomenon, largely caused by anthropogenic activities. Therefore, water quality monitoring and evaluation play prominent roles in safeguarding natural ecosystems, public health, agriculture, and industry. However, exploding populations, agriculture intensification, industrial expansion and rapid urbanization have put tremendous pressures on water resources in terms of quantity and quality [2–4].

Identifying the effects and evaluating water quality are important aspects of water resource protection. Surface water has two pollution modes in surface water [5, 6]: (1) point source (PS) pollution, such as municipal sewage and industrial wastewater; and (2) non-point source (NPS) pollution, such as soil erosion, fertilizer, and pesticides [7]. Because of intensive human activities, regional NPS pollution is unevenly distributed and PS pollution may introduce uncertainty to the correlation of water quality parameters (WQPs) and land-use types (LUTs) [8]. Many studies have modeled NPS pollution based on LUTs. Therefore, land use is generally considered as NPS pollution [9, 10]. Land use reflects the alteration of the natural environment and has a significant impact on water quality [11, 12]. Largely due to pollution dispersion caused by complex interactions between river hydrology and land use pattern, it is extremely difficult to evaluate NPS pollution [3, 13]. Consequently, the relationship between LUTs and WQPs may appear spatially heterogeneous, it varies on different scales [14]. Furthermore, exploring the correlation of LUTs and WQPs is valuable for identifying the main impact factors to water contamination and required for water ecology protection.

Since the 1970s, the impacts of LUTs on WQPs have been studied by many researchers [15, 16] and ascribed water quality degradation to human land uses [15, 17]. Water quality often gets degraded when controls on pollution sources are not enforced [1]. Furthermore, some studies have found that the actual land use deviates from the land use capacity, which leads to the contradiction between the environment and land use, and finally accelerates the deterioration of water quality [18, 19]. Collectively, it can be summarized that anthropogenic activities such as intensive farming, industry and rapid urbanization are found to be the key factors that negatively affect water quality [1, 20, 21]. The proportions of built-up land and farmland have a detrimental impact on water quality [6]. With rapid urbanization, the increase in urban runoff has aggravated NPS pollution [1]. Moreover, forests and grasslands act as net sinks in the cycling of nutrients due to the fixation and adsorption of pollutants [18].

The influences of LUTs on WQPs are highly variable in scale [22]. For example, the spatial scale, i.e., catchment, riparian buffer, and site buffer, has often been used to relevant researches [1, 23]. However, the understanding of these studies has not yet been unified, and the spatial scale with the strongest influence on WQPs has not been clarified. Although the above studies have attracted considerable attention since the 1970s [24, 25], many unanswered questions remain [26]. For example, the multiscale impacts of LUTs on WQPs are still highly controversial. Several studies have reported that LUTs at the watershed scale explain overall WQPs better than those at the riparian scale [7, 23, 27, 28]. In contrast, other studies have reported that LUTs at the riparian scale can better predict variations in WQPs [1, 29, 30]. These contradictory findings are likely due to the unique geographical characteristics and highly dynamic aquatic ecosystems in each research area and differences in data materials and research approaches [31].

In addition, previous study areas were generally considered as a watershed unit composed of dendritic rivers [25]. However, defining the river grade and catchment boundary in a reticular river network area (RRNAs) is difficult because of the dense and complex network structure [32]. Consequently, the impact of LUTs on WQPs may differ in different areas [26, 33]. In recent years, human activities have seriously disturbed the RRNAs, and the water quality in RRNAs is more sensitive to local land use than that in other types of areas [25]. With the advancement of PS pollution control, NPS pollution has become the main factor affecting the water quality in RRNAs [25]. Since it is difficult to determine river grades and watershed boundaries in RRNAs, the impact of LUTs on WQPs may vary considerably from region to region [25, 33, 34].

Following from the above analysis, this study focuses on the Liyang Section of the Nanxi River System (Liyang City), a typical RRNAs in eastern China, as a case study. The research used the stepwise multiple linear regression (SMLR) and redundancy analysis (RDA) methods to demonstrate how land use affected water quality, both spatially and seasonally. Specifically, the main aims of the study mainly are to: (1) characterize seasonal and spatial variations of water quality using Kruskal–Wallis test; (2) explore differences in land use metrics at multiple buffer scales; (3) reveal the seasonal differences in land-use impacts on WQPs based on RDA and SMLR; and (4) analyze the scale effects of the spatiotemporal variability in the correlation between WQPs and LUTs.

## Materials and methods

### Study area

**Fig 1:**
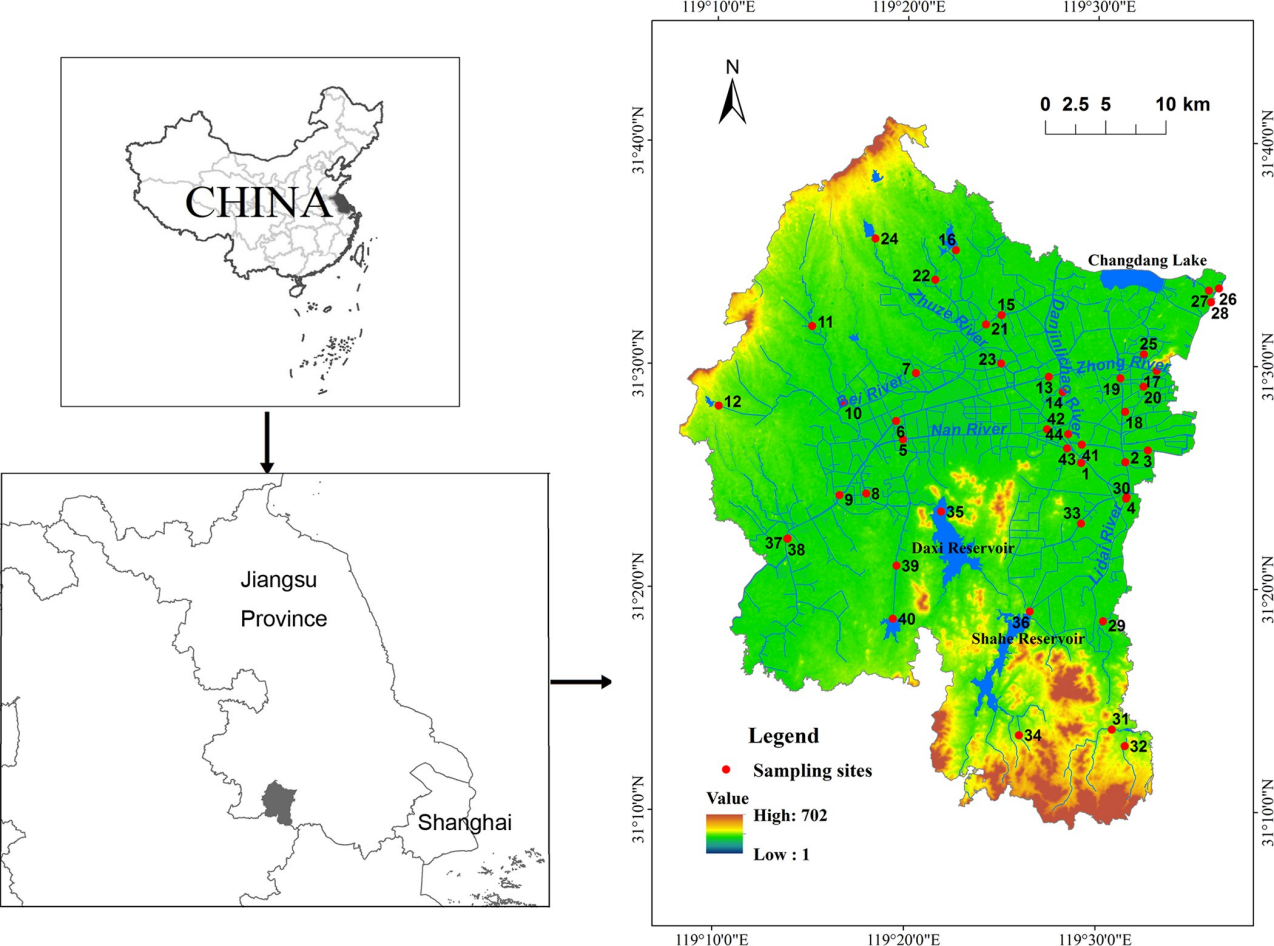
Spatial distribution of the sampling sites and water system in the study area.

Liyang City (119°08'-119°36'E, 31°1'-31°41'N) is located in the southwestern part of Jiangsu Province, China (Fig 1), and its total area is 1535.87 km^2^. The study area is located in an area experiencing a subtropical monsoon climate, with an average annual temperature of 16°C and average annual precipitation of 1147 mm, 70% of which falls between the months of May to September. The topography is characterized by low mountains, hills, and plain polder areas, and the elevation ranges from 1 to 702 m. The soil types in the study area are dominated by paddy soils, yellow brown soils, and yellow cinnamon soils. The natural vegetation in the study area is dominated by evergreen and deciduous broadleaf mixed forest. The main agricultural crops are rice grains, rape, tea, and mulberries. In late 2018, the resident population was 763300, the urban population was 461100, and the urbanization rate was 60.41%.

The study area belongs to the Nanxi River system, which is the main tributary of the Taihu Lake Basin. The main large and medium-sized reservoirs in the territory are the Shahe Reservoir and Daxi Reservoir, the main lakes are Changdang Lake, and the main river channels are the Danjinlicao River, Zhong River, Bei River, Nan River, Zhuze River and Lidai River (Fig 1).

### Water quality parameters

Water quality monitoring data were acquired from our partner agency, the Liyang Environmental Monitoring Center (Liyang, China). We established 44 sampling sites to monitor WQPs (Fig 1 and S1 Table). Water samples were collected once a quarter (March, June, September and December) from 2016–2018. June and September are wet seasons, and March and December are dry seasons. The mean values of WQPs in the wet season and dry season were obtained. Before sampling, 5 L polyethylene bottles were washed with deionized water, dried, and sealed. Then, water samples were collected at depths of 0.5 and 1 m [1]. The water samples were immediately filtered through a Whatman GF/C glass fiber filter (Whatman Ltd, Kent, UK), placed in a low temperature incubator (4°C), and transported them to the laboratory within 48 h. The selected WQPs were ammonia nitrogen (NH_3_-N, mg·L^-1^), total phosphorus (TP, mg·L^-1^), permanganate index (COD_Mn_, mg·L^-1^), and dissolved oxygen (DO, mg·L^-1^). The water samples at depths of 0.5 and 1 m were combined before analysis. All water sample analyses were completed at the Liyang Environmental Monitoring Center (Liyang, China) via the following methods: NH_3_–N, the spectrophotometric method with salicylic acid; TP, the spectrophotometric method; COD_Mn_, acidic potassium permanganate method; and DO, the membrane electrode method.

### Land use data

**Fig 2:**
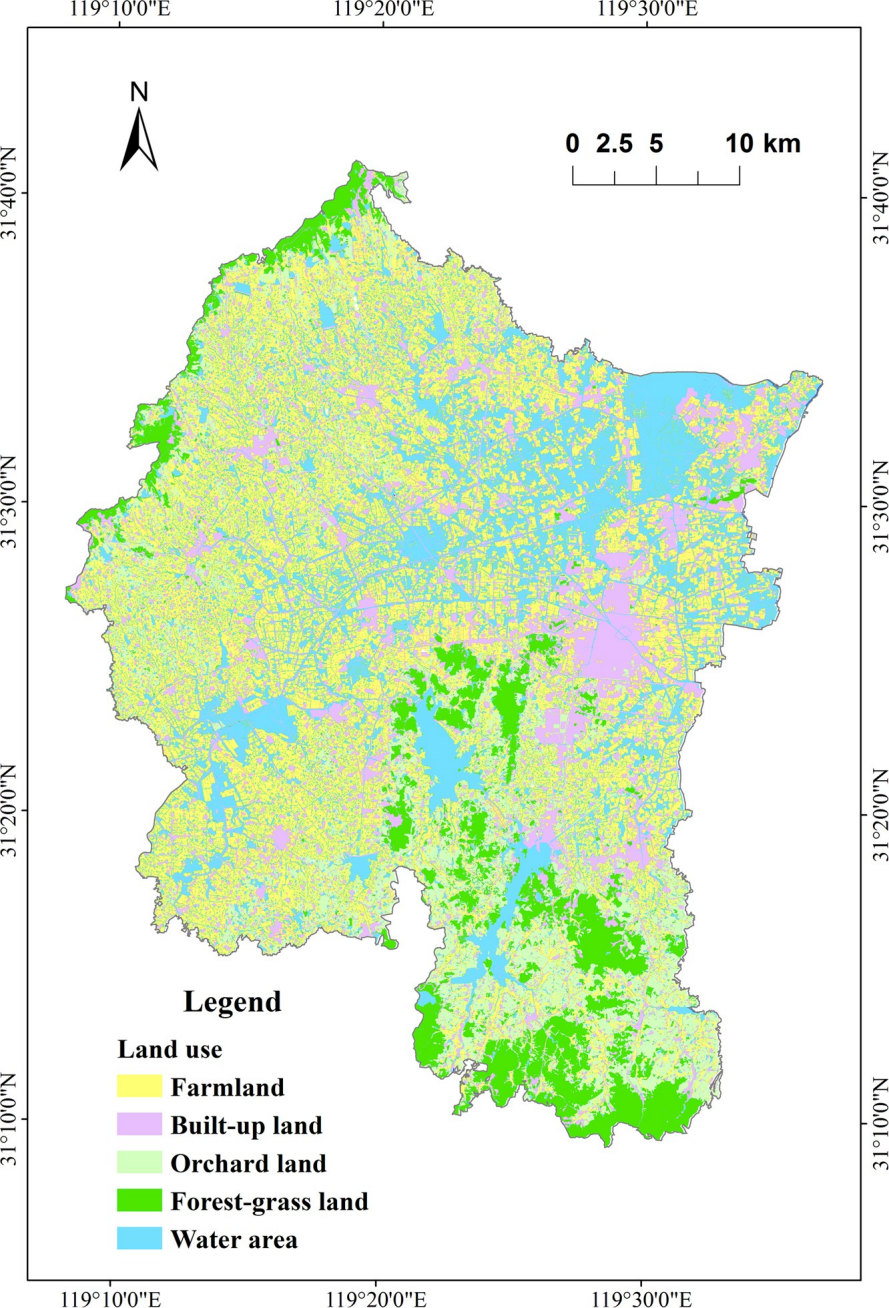
Spatial distribution pattern of LUTs in the study area.

Remote sensing image data has been widely used in land use classification and urban environmental planning [35–42]. The land use data were generated from Landsat 8 images as well as Google Earth images [11]. The Landsat 8 images for 2016 (cloud cover was 0.23%) were acquired from the Geospatial Data Cloud (http://www.gscloud.cn). The remote sensing images were also rectified using field survey data and aerial photographs [43]. The land use data were classified by the maximum likelihood classification (MLC) using ENVI 5.1 software (Exelis Visual Information Solutions, USA). The overall accuracy and the kappa coefficient of the classification were 82.6% and 0.81, separately. Six LUTs were established: viz. farmland, orchard land, built-up land, forest-grassland, water area, and other land (Table 1). The spatial distribution pattern of LUTs is shown in Fig 2.

**Table 1:** Descriptions of the LUTs.

LUTs	Description
Farmland	Paddy field, irrigated land, and rainfed cropland.
Orchard land	Orchard, tea plantation, and other orchard land.
Built-up land	Industrial land, commercial land, residential land, mining land, roads, and public land.
Forest-grassland	Forestland and grassland.
Water area	Rivers, lakes, and ponds.
Other land	Land uses other than those mentioned above, e.g., vacant land, ridges, and bare land.

LUTs represents land-use types.

### Definition of multiple buffer scales

**Fig 3:**
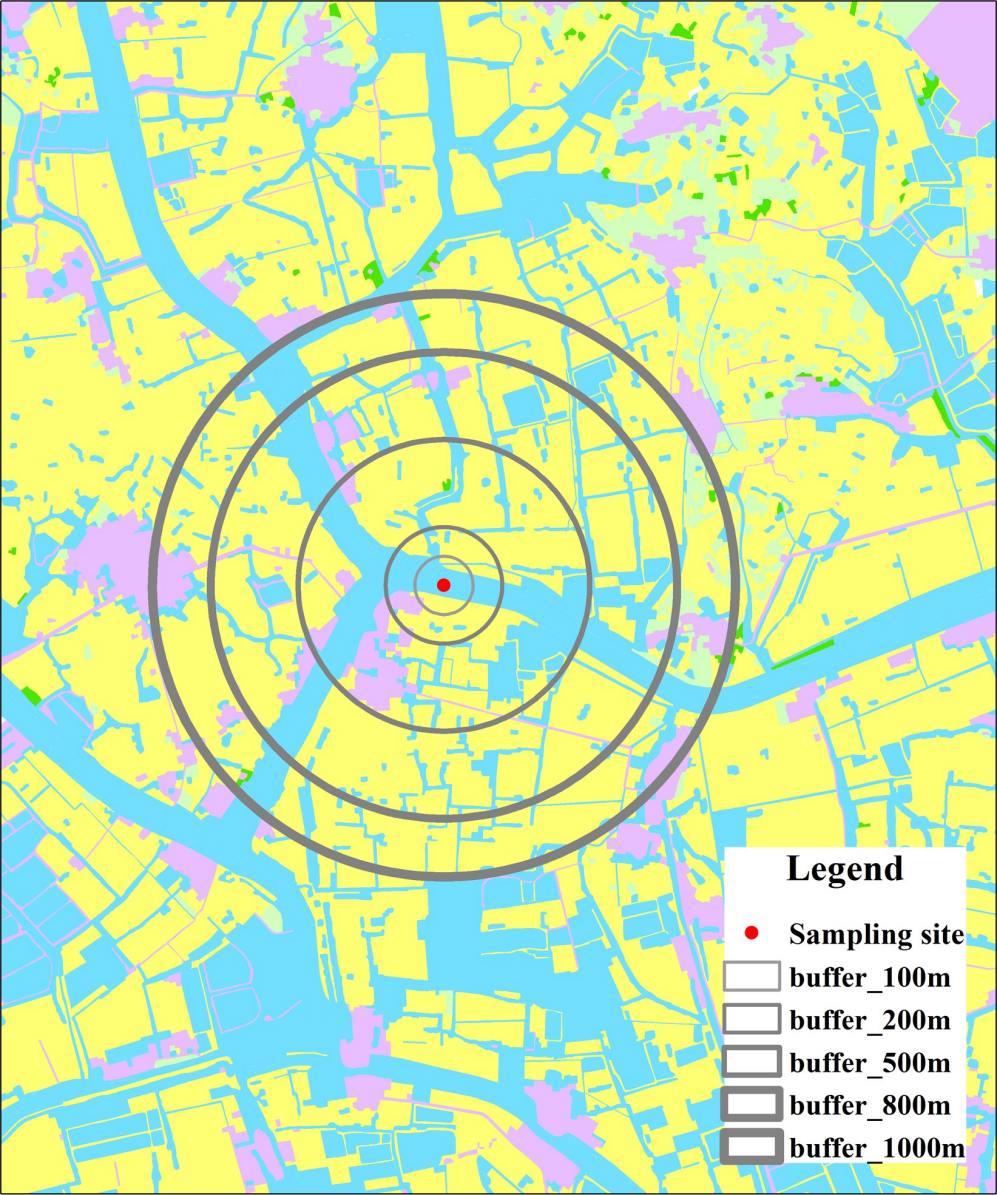
Diagram of the definition of the multiple buffer scales in the typical reticular river network area.

Liyang City is located on the alluvial plain of the Yangtze Delta in East China. It is a typical reticular river network area with dense waterways and a complex network structure. For most rivers, especially small ones, the river flow is slow and bidirectional due to the low gradient of the waterways and the interference of the water gates [32]. Therefore, each water quality sampling site not only represents the upper and lower reaches of its location, but also that of the nearby rivers. The WQPs measured at a sampling site should also be related to the distribution of LUTs and pollution sources in the surrounding areas. Because of the above hydrological characteristics of the river network, it is extremely difficult to define the structure, boundary, and flow sequence of the network [25]. To study the correlation of LUTs and WQPs in the given area, sampling sites were set as geographical centers and a series of buffers were created at five spatial scales (100, 200, 500, 800 and 1000 m) to determine the boundaries of hydrological units and detect scale effects [44, 45] (Fig 3). The area percentage of each LUT in the five buffer scales was then calculated for each sampling site as the land-use indicator. These spatial calculations were performed using ArcMap 10.1 (ESRI Company, USA).

In this study, 100 m was defined as the minimum scale because WQPs are most directly influenced by LUTs at this scale [6] and 1000 m was defined as the maximum buffer scale because many buffers begin to overlap when the buffer size is larger than this threshold [25].

### Statistical analysis

Water quality parameters were tested for their normality (i.e., whether they conform to a normal distribution or not) based on the Kolmogorov–Smirnov test [46]. Because not all WQPs were normally distributed, the differences of WQPs in the dry and wet seasons were determined by the Kruskal–Wallis test [47]. Water quality was evaluated with the exceeding rate (ERs) according to the Grade III environmental quality standards for surface water (GB3838-2002). The ERs is the ratio of the number of times a pollutant exceeds the discharge standard to the total number of pollutants detected:

$$D_{i}=F_{i}/N\times 100\%$$ (1)

where *D*_*i*_ is the exceeding rate of pollutant *i*, *F*_*i*_ is the number of times that pollutant *i* exceeds the standard value of Grade III, and *N* is the total number of times that pollutant *i* is detected.

First, water quality data were analyzed via a detrended correspondence analysis (DCA). Because the maximum value of the lengths of the gradient in the four gradient axes was less than three, the linear model was recommended. Therefore, RDA was selected to explore the impacts of LUTs on all WQPs [43]. Before performing the RDA, log-transformations (ln(x+1)) of all water quality parameters were performed [25, 48, 49]. The RDA was performed to (1) explain the variation (%) in all WQPs that was explained by multiple land use variables; and (2) generate ordination diagrams (biplots), which revealed the correlation of LUTs and WQPs [7, 23]. RDA was executed by CANOCO 5.0 (Microcomputer Power Company, USA).

**Fig 4:**
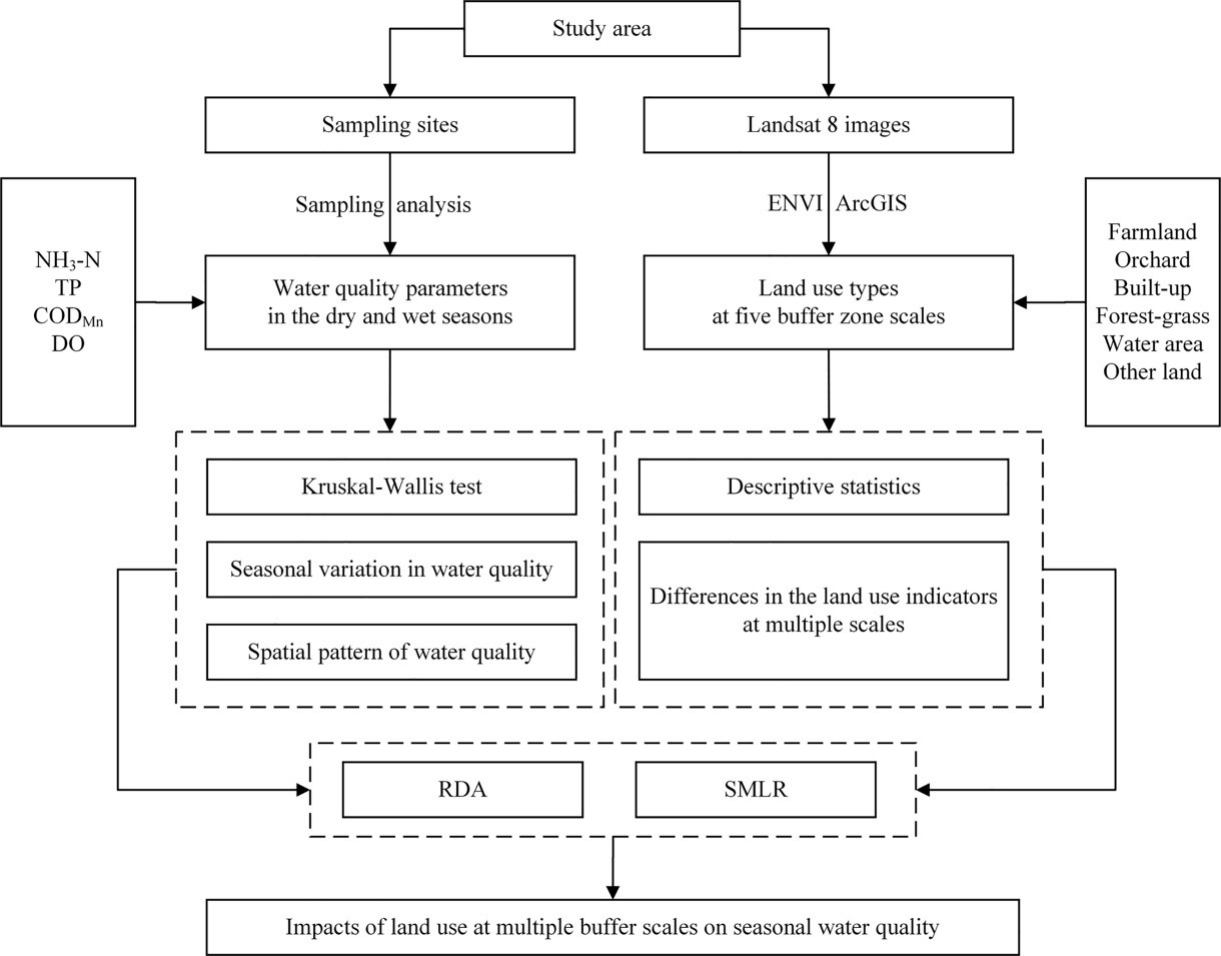
Comprehensive flowchart of the methodology.

Finally, the SMLR was utilized to reveal the correlation of LUTs and WQPs [46]. The SMLR was executed with SPSS 20.0 (IBM Company, USA). A comprehensive flowchart describing the methodology is shown in Fig 4.

## Results

### Seasonal and spatial variations of WQPs

**Fig 5:**
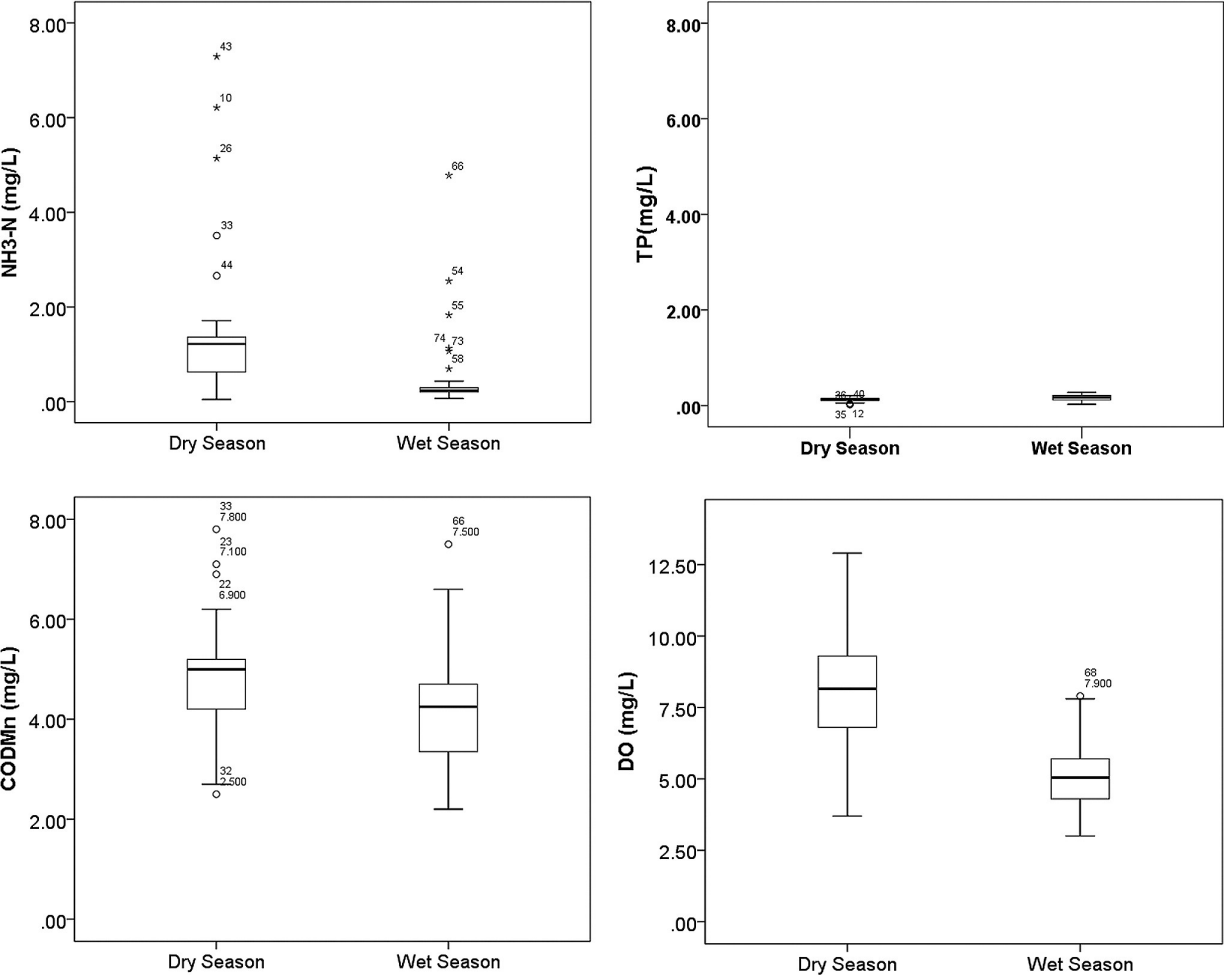
Boxplots of WQPs during the dry and wet seasons.

The seasonal variations of WQPs were significant (Table 2; Fig 5). The Kruskal–Wallis nonparametric test results indicated that NH_3_-N, TP, COD_Mn_, and DO showed obvious seasonal variations (*p* < 0.01). The concentration values of NH_3_-N and COD_Mn_ were significantly higher in the dry season than the wet season, while TP showed the opposite pattern. The DO level was higher in the dry season, and the NH_3_-N value in the dry season was three times that in the wet season. The ERs of NH_3_-N and COD_Mn_ in the dry season were higher than those in the wet season, while that of TP and DO showed the opposite pattern. The ERs of NH_3_-N (63.64%) in the dry season and TP (31.82%) and DO (34.09%) in the wet season were significantly high (Table 2).

**Table 2:** Summary of the seasonal variations of WQPs between the dry and wet seasons.

WQPs (Unit)	Dry season	Wet season	Kruskal–Wallis		Grade III Standard	Exceeding rate (%)	
	Mean ± S.D.	Mean ± S.D.	*H*	*P*		Dry season	Wet season
NH_3_-N (mg·L^-1^)	1.37 ± 1.51	0.47 ± 0.81	14.14	0.000**	≤1.0	63.64	11.36
TP (mg·L^-1^)	0.12 ± 0.05	0.15 ± 0.07	8.17	0.004**	≤0.2	9.09	31.82
COD_Mn_ (mg·L^-1^)	4.76 ± 1.14	4.17 ± 1.17	7.51	0.006**	≤6	9.09	6.82
DO (mg·L^-1^)	8.28 ± 2.08	5.24 ± 1.25	40.44	0.000**	≥5	2.27	34.09

Mean represents the mean value; S.D. represents standard deviation.

*H*, represents the H-Test statistics of the Kruskal–Wallis test.

*, *p* < 0.05

**, *p* < 0.01.

**Fig 6:**
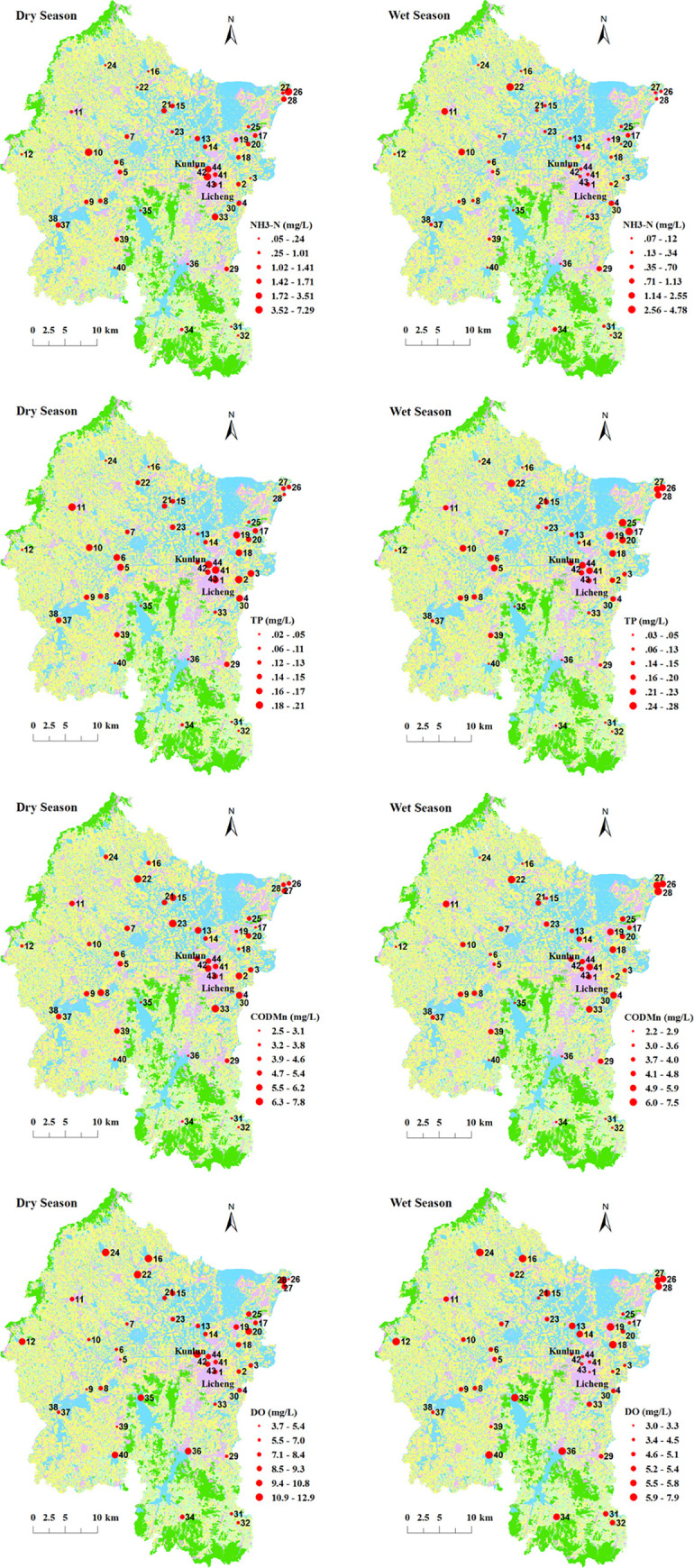
Spatial differences in seasonal WQPs.

Except for TP, most WQPs showed significant spatial differences (Fig 6). The highest values of NH_3_-N (7.29 mg·L^-1^) and COD_Mn_ (7.8 mg·L^−1^) were found at sites 43 and 33, respectively, and the lowest DO value (3 mg·L^-1^) was found at site 1, where the town of Licheng and community of Kunlun are located. These areas have relatively higher levels of urbanization (Fig 6). An increase in impervious surfaces in urbanized areas leads to an increase in surface runoff, and more pollutants are washed into water bodies, which aggravates contamination. The lowest value of NH_3_-N (0.048 mg·L^-1^) was found at sites 35, the lowest value of COD_Mn_ (2.2 mg·L^−1^) was found at sites 32 and 34, and the highest DO value (12.9 mg·L^-1^) was found at site 22. These areas are nature reserves such as reservoir and mountain forest (Fig 6). However, TP was high in wet and dry seasons, which may be related to urban domestic sewage and the overuse of chemical fertilizers.

### Differences in LUTs

**Fig 7:**
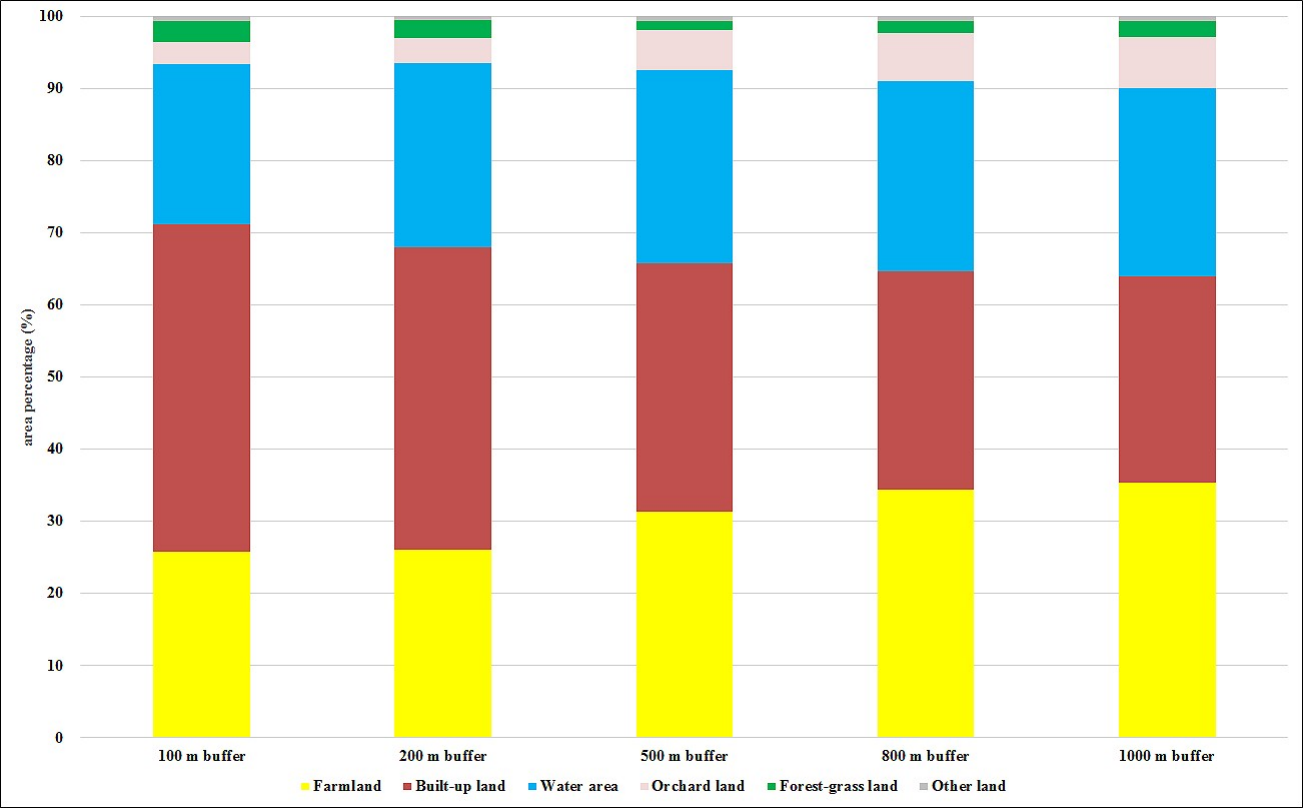
Proportional areas of LUTs at the buffer scales within each radius.

Farmland, built-up land, and water area were the major LUTs at all buffer scales (Table 3; Fig 7), with farmland covering 25.76% (100 m buffer) to 35.37% (1000 m buffer), built-up land covering 28.61% (1000 m buffer) to 45.44% (100 m buffer), and water area covering 22.16% (100 m buffer) to 26.77% (500 m scale) of their respective land use areas. In the whole region, farmland accounted for 38.96%, water area accounted for 22.75%, built-up land accounted for 16.20%, and the total was nearly 80%. Regardless of the spatial scale, other land occupied the smallest proportion (<0.65%); therefore, other land was excluded from subsequent analyses. As the spatial scale increased, the proportions of farmland and orchard land increased while the proportion of built-up land decreased (Table 3; Fig 7).

**Table 3:** Changes in land use proportions throughout the whole region and at 100–1000 m buffer scales in the study area.

Land use	Whole region	Mean ± S.D.				
		100 m buffer	200 m buffer	500 m buffer	800 m buffer	1000 m buffer
Farmland (%)	38.96	25.76 ± 26.79	26.07 ± 21.00	31.35 ± 18.89	34.35 ± 17.21	35.37 ± 16.38
Built-up land (%)	16.2	45.44 ± 34.34	41.97 ± 28.63	34.42 ± 25.27	30.37 ± 22.68	28.61 ± 21.51
Water area (%)	22.75	22.16 ± 21.19	25.44 ± 16.37	26.77 ± 16.73	26.30 ± 15.00	26.04 ± 13.98
Orchard land (%)	12.2	3.01 ± 7.21	3.48 ± 5.84	5.33 ± 10.52	6.61 ± 13.00	7.04 ± 13.14
Forest-grassland (%)	9.8	2.97 ± 12.76	2.49 ± 10.15	1.32 ± 3.42	1.78 ± 3.50	2.29 ± 4.42
Other land (%)	0.09	0.64 ± 0.19	0.58 ± 0.11	0.59 ± 0.40	0.61 ± 0.25	0.65 ± 0.17

Mean represents the mean value; S.D. represents the standard deviation.

### Land use impacts on seasonal WQPs

The LUTs from 100 m to 1 km buffer scales and seasonal WQPs were analyzed via RDA. Overall, 30% and 20% of WQPs during the dry and wet season, respectively, were explained by the first two axes (Table 4). The first axis showed a pollution gradient (e.g., TP decreased along the axis) that was positively correlated with built-up land and negatively correlated with forest-grassland. The primary explanatory variable was orchard land (38.8%–54.4%) at multiple buffer scales. The secondary important explanatory variables were forest-grassland (17.6%–28.8%) and farmland (22.8%) at the 100–500 m buffer scales and water area (28.6%–34.9%) at the 800–1000 m buffer scales (Table 4).

**Table 4:** RDA results for percentages of WQPs explained by LUTs.

Seasons	Buffer scales	Explained variation (%)			Pseudo-F	*p-*value	Explanatory variables (%)
		Axis 1	Axis 2	All axes			
Dry	100 m buffer	29.16	1.73	31.54	3.5	0.010*	Orchard (39.9), Farmland (22.8)
	200 m buffer	26.38	2.35	29.13	3.1	0.016*	Orchard (54.4), Forest-grass (20.5)
	500 m buffer	23.41	2.80	27.22	2.8	0.026*	Orchard (54.0), Forest-grass (17.6)
	800 m buffer	23.00	2.84	27.35	2.9	0.024*	Orchard (52.1), Water (30.0)
	1000 m buffer	25.09	3.16	30.10	3.3	0.014*	Orchard (51.3), Water (34.9)
Wet	100 m buffer	12.38	3.45	16.35	1.5	0.17	Orchard (39.4), Forest-grass (28.8)
	200 m buffer	11.74	4.00	16.68	1.5	0.132	Orchard (38.8), Forest-grass (28.0)
	500 m buffer	14.10	3.02	17.84	1.7	0.108	Orchard (53.4), Forest-grass (20.6)
	800 m buffer	15.59	3.75	20.58	2.0	0.070	Orchard (49.7), Water (28.6)
	1000 m buffer	17.28	4.49	23.43	2.3	0.034*	Orchard (45.9), Water (29.8)

*, *p* < 0.05

**, *p* < 0.01.

The RDA results revealed that the correlations of LUTs and WQPs had significant seasonal variations. The explained variations of all axes for WQPs were >27.0% during the dry season, and this percentage decreased by 6.67%–15.19% in the wet season (Table 4). The explained variation in all axes initially decreased and then increased during the dry season but increased continuously during the wet season (Table 4).

**Fig 8:**
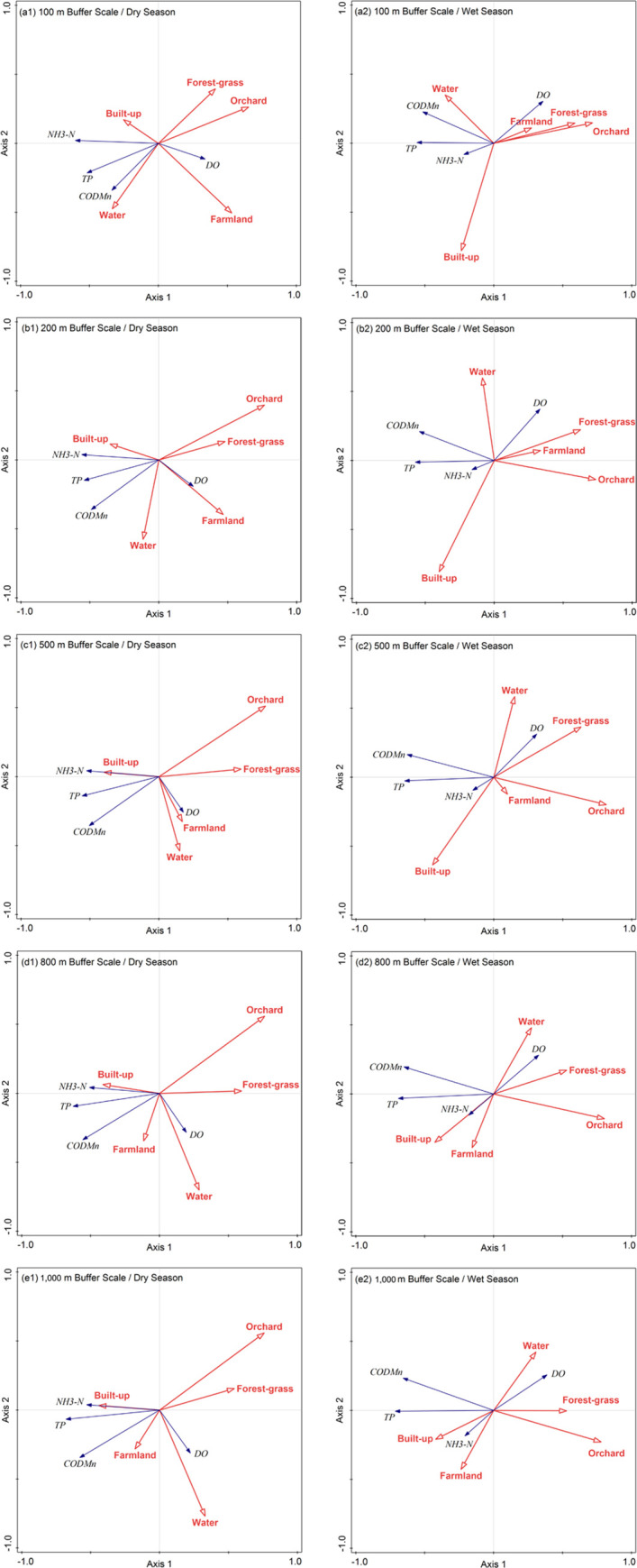
Correlations between LUTs and WQPs according to RDA.

LUTs had multi-scale effects on WQPs (Fig 8). At all buffer scales, built-up land and DO had negative correlation while built-up land was positively correlated with NH_3_-N, TP, and COD_Mn_, with correlations in the dry season being greater than that in the wet season. Orchard land and forest-grass land were negatively correlated with NH_3_-N, COD_Mn_ and TP, while positively correlated with DO. In particular, the correlation of orchard land was more significant than that of forest-grassland. Farmland and DO had negative correlation while Farmland was positively correlated with TP, NH_3_-N, and COD_Mn_ at the 800–1000 m buffer scales.

The SMLR results indicated that orchard land had the greatest impact on water quality (Tables 5 and 6). Orchard land and NH_3_-N in the dry season had negative correlation, while COD_Mn_ and TP in dry and wet seasons had negative correlation with orchard land.

**Table 5:** SMLR results for the impacts of LUTs on WQPs at each buffer scale during the dry season.

	Farmland	Orchard land	Built-up land	Forest-grassland	Water area	Adjusted *R*^*2*^
100 m buffer scale						
NH_3_-N		-				0.254
TP		-	+			0.396
COD_Mn_		-		-	+	0.283
DO						
200 m buffer scale						
NH_3_-N		-				0.266
TP		-	+			0.434
COD_Mn_		-		-		0.330
DO						
500 m buffer scale						
NH_3_-N		-				0.298
TP		-			-	0.600
COD_Mn_				-		0.263
DO						
800 m buffer scale						
NH_3_-N		-				0.148
TP		-			-	0.613
COD_Mn_		-				0.326
DO						
1000 m buffer scale						
NH_3_-N		-				0.245
TP	+	-	+		-	0.671
COD_Mn_	+	-				0.408
DO						

+ represents a positive correlation;—represents a negative correlation; no symbol indicates no correlation.

**Table 6:** SMLR results for the impacts of LUTs on WQPs at each buffer scale during the wet season.

	Farmland	Orchard land	Built-up land	Forest-grassland	Water area	Adjusted *R*^*2*^
100 m buffer						
NH_3_-N						
TP		-				0.186
COD_Mn_		-		-		0.201
DO			-			0.327
200 m buffer scale						
NH_3_-N						
TP		-	+			0.274
COD_Mn_		-		-		0.248
DO			-			0.293
500 m buffer scale						
NH_3_-N						
TP		-			-	0.451
COD_Mn_		-				0.247
DO			-			0.190
800 m buffer scale						
NH_3_-N						
TP		-			-	0.520
COD_Mn_		-				0.268
DO			-			0.170
1000 m buffer scale						
NH_3_-N						
TP		-			-	0.540
COD_Mn_		-				0.306
DO			-			0.182

+ represents a positive correlation;—represents a negative correlation; no symbol indicates no correlation.

The forecast directions of water area and forest-grassland were similar to that of orchard land. Forest-grassland and COD_Mn_ had negative correlation, while water area and TP had negative correlation. However, their forecast levels were not as good as that of orchard land.

Built-up land appeared in many of the SMLR models and was frequently positively correlated with parameters related to polluted water quality. For example, built-up land and TP had positive correlation in the dry season, while built-up land and DO had negative correlation in the wet season.

Surprisingly, farmland was not a major predictor of degraded water quality [30, 50]. COD_Mn_ and TP were positively correlated with farmland only at the 1 km buffer scale during the dry season.

According to the adjusted *R*^*2*^ trend, the ability of LUTs to predict WQPs increased as the buffer scale increased except for DO. However, the land use indicators did not show any predictive ability for DO during the dry season or NH_3_-N during the wet season (adjusted *R*^*2*^ = 0).

## Discussion

### Correlations of LUTs and WQPs

This research revealed that forest-grassland had positive effects on WQPs and built-up land had negative effects on WQPs (Fig 8; Tables 5 and 6). These results were consistent with those of most previous studies [43, 51–53]. The highest NH_3_-N, COD_Mn_, and TP concentrations most frequently appeared in urbanized areas (Fig 6), indicating that built-up land was the main source of PS and NPS pollution. Rapid urbanization has increased the impervious surface area, which has greatly increased surface runoff volumes. Urban storm runoff transports relatively higher amounts of pollutants (e.g., domestic sewage and waste discharge) into rivers, which increases the nutrient concentrations in surface waters [43]. Vegetation cover is the primary factor that intercepts NPS pollution, and most water quality parameters are negatively associated with forest-grassland due to reductions of surface runoff and soil erosion [5].

The ability of orchard land to predict nutrients was somewhat surprising. In all seasons and at all buffer scales, orchard land was significantly negatively correlated with most WQPs (Fig 8; Tables 5 and 6). This result was not consistent with other studies [5, 54–56] and might be related to the vigorous promotion of ecological agriculture; the strict control of pesticide use in woodlands, tea gardens, orchards, and grassland; and the increased promotion of organic fertilizers, biological pesticides, and attractants. Therefore, orchard land could have a favorable impact on improving WQPs similar to that of ecological forests.

The SMLR results revealed that the ability of farmland to predict water pollution was not obvious (Fig 8; Tables 5 and 6). This result was not consistent with those of other studies [57, 58] but was similar to another research conclusion [43], which was likely because of the effect of decreased water quality in farmland due to built-up land, which corresponds to major PS pollution and rapid urbanization [43]. In addition, the implementation of agricultural management measures, such as straw returning, increased organic fertilizer application, less tillage and no-tillage practices, crop-bean rotations, and the reduction and recycling of agricultural film in the study area resulted in reduced pollutants in the cultivated soil. Farmland and nutrients had positive correlation only at the relatively greater buffer scale.

### Seasonal differences in LUTs impacts on WQPs

The RDA results indicated that all WQPs were better explained by LUTs during the dry season than the wet season (Table 4), and the adjusted *R*^*2*^ values in the SMLR were also generally greater during the dry season than the wet season (Tables 5 and 6). The same conclusion was reported in another study [26]. One explanation for this result is that stream flow reduction in the dry season led to higher concentrations of most nutrients and pollutants. During the dry season, the higher concentration values of pollutants were also related to farming activities, such as plant protection, fertilization, and weeding [7].

The SMLR results showed that farmland had greater negative impacts on contaminants (e.g., TP, COD_Mn_) during the dry season than the wet season. This conclusion is probably related to agricultural activities (e.g., spring ploughing, fertilization, and weeding) during the dry season. Because the stream water quality was affected more by PS pollution from urban areas, built-up land was more greatly related to TP during the dry season. The contaminants were diluted with the increased rainfall and river discharge during the wet season [59, 60]. During the wet season, built-up land and DO had strongly negative correlation because of the large variety of organic pollutants on the urban surface that are washed into the rivers by storm runoff [58].

### Scale effects for the relationships of LUTs and WQPs

As the buffer scale increased, the impact of LUTs on WQPs also increased (Tables 4–6). This finding suggests that a relatively larger spatial scale needs to be considered for regional water quality management [23, 61]. However, other studies have reported that the riparian scale is superior to other larger scales when predicting water quality [1]. These opposite results may be due to the selected LUTs and WQPs [1, 26, 28, 62].

The scale effect of different LUTs was variable. At larger scales, farmland was more closely related to water quality because farmland is widely distributed in the study region (Fig 6). At smaller scales, built-up land had relatively strong effects on water quality [46], which is likely due to the frequent distribution of built-up land along rivers and the serious disturbance of human urbanization activities on river ecosystems [63].

The effects of farmland and water area on WQPs at different buffer scales were complex (Fig 8; Tables 5 and 6). Although farmland is the main NPS pollution source at the catchment scale, farmland soils can also intercept and absorb some nutrients. Agricultural practices such as spraying pesticides and applying fertilizers can transport nutrient pollutants to rivers via farmland runoff; however, the transport of these pollutants is affected by many factors, such as rainfall, runoff, soil properties, and the terrain gradient. In addition, the complexity of predicting water quality by water area may be associated with the regional characteristics of reticular river network areas.

### Suggestions for water resource management

The observed NH_3_-N, TP, and DO levels seriously exceeded the Grade III environmental quality standards for surface water (*D*_*i*_ > 30%), which was largely related to the organic pollutants in domestic sewage in urban residential areas. The results revealed that the impact of a large buffer scale for water quality is slightly greater than that of smaller buffer scales (Tables 4–6), which indicated that water quality management is mainly a regional problem. Water resource management needs to solve problems on a larger scale, such as rational fertilization, livestock control, and poultry breeding. Developing cluster agriculture is also beneficial.

In addition, the impact of leaching or deep drainage on water quality mainly occurs in the low mountains and hilly areas in the south and northwest. The concentration of organic matter and nutrients was reduced by maintaining clustered forests and grassland to reduce the impact of leaching and deep drainage on river water quality [23].

## Conclusions

In this study, the WQPs was affected by land use to a certain degree. In particular, the favorable influence of orchard land was significant. The factors affecting water quality included the season, spatial scale, and selected land use indicators. The effects of LUTs on WQPs were more significant in the dry season than the wet season. The total variation explained by LUTs regarding WQPs was slightly stronger at the 1 km buffer scale. Our study indicated that built-up land had negative effect on WQPs while orchard land had favorable effect on WQPs because of the agricultural management measures. We studied the temporal-spatial impacts of LUTs on WQPs in a RRNAs. The research results provide key insights into the multi-scale impact of LUTs on WQPs and showed the impacts of effective land use on water quality management, thereby providing reference values for the sustainable development of regional water resource. In future research, assessing the comprehensive influence of topography, soil, and geology on regional water quality should be prioritized. It is also recommended that future studies involve landscape patterns and land development intensity as these are expected to further reveal the intricate relationships between human activities, regional economies, and water quality.

## Supporting information

S1 Table Summary of the sampling sites in this study. (DOC)
