# Supplementary material for: Impacts of land use at multiple buffer scales on seasonal water quality in a reticular river network area
Source: PLoS One. 2021 Jan 6;16(1):e0244606. doi: 10.1371/journal.pone.0244606 (PMC7787537; doi:10.1371/journal.pone.0244606)
Supplement: S1 Table — (DOC) [file pone.0244606.s001.doc]

**S1 Table.** Summary of the sampling sites in this study

| ID | Name | Town (Community) | River | Longitude | Latitude |
| --- | --- | --- | --- | --- | --- |
| 1 | Xia Bridge | Licheng Town | Danjinlichao River | 119.484434 | 31.424723 |
| 2 | Zhangxiang Bridge | Licheng Town | Wutai Canal | 119.524387 | 31.428303 |
| 3 | Taiping Bridge | Licheng Town | Madian River | 119.544267 | 31.436922 |
| 4 | Xincun Lane | Licheng Town | Danjinlichao River | 119.525996 | 31.402084 |
| 5 | Nandu Bridge | Nandu Town | Nan River | 119.330393 | 31.444481 |
| 6 | Zhongdu Bridge | Nandu Town | Zhong River | 119.324208 | 31.458338 |
| 7 | Songjia Bridge | Nandu Town | Bei River | 119.341390 | 31.494011 |
| 8 | Qianliu Bridge | Nandu Town | Daxi River | 119.298652 | 31.403744 |
| 9 | Qiankou | Shangxing Town | Nan River | 119.275161 | 31.402516 |
| 10 | Tangdong Bridge | Shangxing Town | Bei River | 119.284347 | 31.470198 |
| 11 | Shangxingjie Bridge | Shangxing Town | Shangxing River | 119.250375 | 31.528722 |
| 12 | Zhulin reservoir | Shangxing Town | Zhulin reservoir | 119.169388 | 31.467928 |
| 13 | Sanyi Bridge | Bieqiao Town | Zhong River | 119.457452 | 31.491793 |
| 14 | Zhulian Bridge | Bieqiao Town | Danjinlichao River | 119.469757 | 31.480519 |
| 15 | Choumiu Bridge | Bieqiao Town | Bei River | 119.416066 | 31.537723 |
| 16 | Tangma reservoir | Bieqiao Town | Tangma reservoir | 119.376755 | 31.586103 |
| 17 | Shanqian Bridge | Daitou Town | Zhong River | 119.551632 | 31.496223 |
| 18 | Xiaowei Lane | Daitou Town | Zhaocun River | 119.523695 | 31.466107 |
| 19 | Dainan Bridge | Daitou Town | Zhaocun River | 119.519934 | 31.490634 |
| 20 | Dadongdang | Daitou Town | Changzhou River | 119.540590 | 31.484504 |
| 21 | Yangjiawan | Zhuze Town | Bei River | 119.402467 | 31.530749 |
| 22 | Wangjiacun Highway Bridge | Zhuze Town | Tuobanqiao River | 119.357615 | 31.563751 |
| 23 | Yu Bridge | Zhuze Town | Zhuze River | 119.415787 | 31.501379 |
| 24 | Lvzhuang reservoir | Zhuze Town | Lvzhuang reservoir | 119.307232 | 31.593963 |
| 25 | Poxu Bridge | Shanghuang Town | Huadang River | 119.540442 | 31.508606 |
| 26 | Xinshi Bridge | Shanghuang Town | Zhonggan River | 119.605995 | 31.558018 |
| 27 | Shanxia Bridge | Shanghuang Town | Zhonggan River | 119.596978 | 31.556790 |
| 28 | Langgan Bridge | Shanghuang Town | Pugu River | 119.598882 | 31.548003 |
| 29 | Daibu Bridge | Daibu Town | Lidai River | 119.505908 | 31.308338 |
| 30 | Changmu Bridge | Daibu Town | Lidai River | 119.531035 | 31.400041 |
| 31 | Bailiugan Bridge | Daibu Town | Qingxi River | 119.513438 | 31.228714 |
| 32 | Wanli Bridge | Daibu Town | Lijiayuan River | 119.525175 | 31.216137 |
| 33 | Wangxian Bridge | Tianmuhu Town | Guxian River | 119.486142 | 31.382224 |
| 34 | Pingqiao Bridge | Tianmuhu Town | Pingqiao River | 119.433122 | 31.223788 |
| 35 | Daxi reservoir | Tianmuhu Town | Daxi reservoir | 119.365339 | 31.390938 |
| 36 | Shahe Reservoir | Tianmuhu Town | Shahe Reservoir | 119.446578 | 31.313425 |
| 37 | Chengmawei | Shezhu Town | Nan River | 119.231085 | 31.369414 |
| 38 | Hekou Bridge | Shezhu Town | Nan River | 119.229912 | 31.369798 |
| 39 | Zhoucheng Bridge | Shezhu Town | Zhoucheng River | 119.325334 | 31.349939 |
| 40 | Qiansong Reservoir | Shezhu Town | Qiansong Reservoir | 119.328003 | 31.307265 |
| 41 | Yinwu Bridge | Kunlun community | Beihuan River | 119.486567 | 31.441133 |
| 42 | Hongkou Bridge | Kunlun community | Zhuze River | 119.456366 | 31.452034 |
| 43 | Laijiang Bridge | Kunlun community | Nan River | 119.473779 | 31.438235 |
| 44 | Zhengchanglu Bridge | Kunlun community | Danjinlichao River | 119.474514 | 31.448853 |
